# Ferroptosis of Immune Cells in Infection, Inflammation and Tumor Progression

**DOI:** 10.3390/biom15101464

**Published:** 2025-10-16

**Authors:** Hanxiao Xu, Yanjun Lu, Qingwei Zeng, Xudong Zhu, Wenxian Guan, Song Liu

**Affiliations:** Division of Gastric Surgery, Department of General Surgery, Nanjing Drum Tower Hospital, Affiliated Hospital of Medical School, Nanjing University, Nanjing 210008, China; 201230055@smail.nju.edu.cn (H.X.);

**Keywords:** ferroptosis, immune cells, infection, inflammation, tumor

## Abstract

Ferroptosis has been described as a unique form of programmed cell death. Its mechanism mainly involves iron metabolism disorders, lipid peroxidation and impaired antioxidant defense systems. The role of ferroptosis in infection, inflammation and tumor development has gained widespread attention. In particular, ferroptosis in immune cells significantly affects immune responses during disease progression. This review aims to summarize the advanced research progress of ferroptosis in different types of immune cells. It also discusses potential therapeutic targets and strategies to regulate ferroptosis, which may offer novel interventions for diseases, especially cancer.

## 1. Introduction

Ferroptosis is a distinct form of non-apoptotic programmed cell death. It is iron-dependent and characterized by the accumulation of lipid peroxides. Ferroptosis was first coined by Dixon et al. in 2012 [1]. Since its first description, an increasing number of studies have revealed its involvement in the development and progression of various diseases, including infections, inflammatory conditions and cancers [2].

The primary mechanism underlying ferroptosis involves the disruption of iron homeostasis and the subsequent excessive accumulation of lipid reactive oxygen species (ROS) mainly via Fenton reaction. These ROS subsequently interact with polyunsaturated fatty acids (PUFAs) and trigger lipid peroxidation, causing membrane damage and ultimately resulting in ferroptosis [1,3]. The main factors that trigger ferroptosis are: (a) impairment of the antioxidant defense systems, including the inhibition of glutathione peroxidase 4 (GPX4)/glutathione (GSH) system and non-GPX4-dependent antioxidant systems like ferroptosis suppressor protein 1 (FSP1)-ubiquinone or coenzyme Q10 (CoQ10)-NAD(P)H pathway and GTP cyclohydrolase-1/tetrahydrobiopterin (GCH1/BH4) pathway, (b) increased activation and incorporation of PUFAs into membrane lipids involving enzymes such as acyl-coenzyme A synthetase long-chain family member 4 (ACSL4) or lysophosphatidylcholine acyltransferase 3 (LPCAT3), and (c) disruption of iron uptake, transport, storage and metabolism [4,5,6,7].

Ferroptosis exerts complex impacts on disease processes especially in cancer, where it exhibits a dual role in the tumor microenvironment (TME). On one hand, it can inhibit tumor progression by inducing ferroptosis of tumor cells. On the other hand, ferroptosis-induced inflammatory response and immunosuppression may promote tumor progression [8]. Ferroptosis can affect the activity and function of a variety of immune cells, including neutrophils, macrophages, T lymphocytes, B lymphocytes, natural killer (NK) cells, dendritic cells (DCs) and myeloid-derived suppressor cells (MDSCs). In different disease contexts, immune cells develop various mechanisms to regulate ferroptosis, influencing immune response and disease progression. These mechanisms provide targets for interventional strategies. In addition, various immune cells exhibit differential sensitivity to ferroptosis, indicating that targeted modulation of ferroptosis could enhance immune responses in both cancer and non-cancer contexts. This review aims to explore the mechanisms of ferroptosis in immune cells and its role in tumor progression and immune responses. It will also offer potential intervention strategies to harness these processes for improved disease treatment outcomes.

## 2. Ferroptosis of Immune Cells

### 2.1. Neutrophils

Neutrophils play a significant role in both innate and adaptive immunity. As the first responders to invading pathogens and inflammatory signals, they can quickly migrate to the site of inflammation and infection. Furthermore, they attract and activate other immune cells by releasing substances such as chemokines, cytokines and enzymes [9].

Neutrophils exert their functions mainly through phagocytosis, degranulation and the release of neutrophil extracellular traps (NETs). NETs are web-like structures composed of DNA, histones and a variety of antimicrobial proteins. They participate in immune defense by resisting microorganisms and amplifying inflammatory responses [10,11,12]. Wang et al. discovered that in fluoride-induced brain inflammation, neutrophils underwent ferroptosis, leading to the release of NETs, which aggravated the inflammatory response. The potential mechanism beneath this ferroptosis in neutrophils was that fluoride disrupted calcium homeostasis, and then L-type calcium ion channels opened, allowing free iron to enter the cells [13].

In addition, as the dominant immune cells in the circulation, neutrophils also contribute to various autoimmune diseases. In systemic lupus erythematosus, serum autoantibodies and interferon (IFN) α contribute to neutropenia by triggering neutrophil ferroptosis. During this process, the expression of GPX4 is inhibited due to the activation of the Calcium/calmodulin-dependent protein kinase IV (CaMKIV)/cAMP-responsive element modulator α (CREMα) axis. Autoantigens released from ferroptotic neutrophils stimulate B lymphocytes and DCs to produce additional autoantibodies and Type I IFNs, thus forming a positive feedback loop [14].

**Figure 1:**
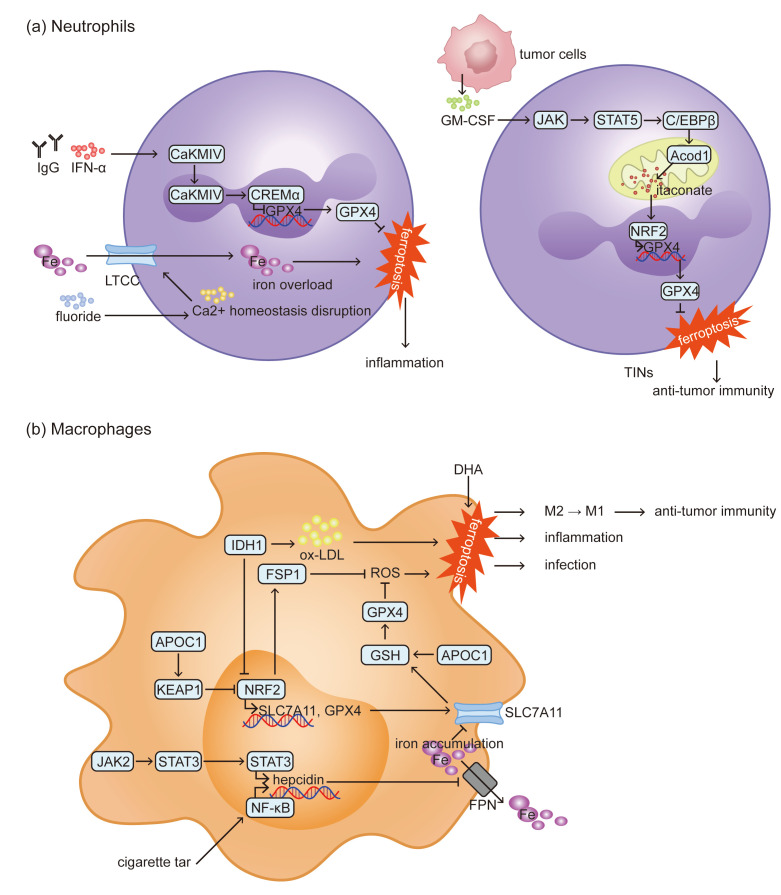
Ferroptosis mechanisms and consequences of neutrophils and macrophages. (**a**) In inflammatory and autoimmune diseases, calcium homeostasis imbalance-induced iron overload and failure of the antioxidant system collectively trigger neutrophil ferroptosis, which exacerbates inflammatory responses. In contrast, within the TME, TINs suppress ferroptosis through metabolic reprogramming, thereby exerting immunosuppressive effects. (**b**) Iron metabolism disorders induced by JAK2 and cigarette tar, as well as the imbalance of the antioxidant system induced by APOC1 and IDH, are involved in ferroptosis in macrophages. In tumors, macrophage ferroptosis promotes their polarization toward the M1 phenotype, thereby enhancing anti-tumor immunity. However, in inflammatory and infectious diseases, macrophages ferroptosis promotes disease progression.

Tumor-infiltrating neutrophils (TINs) are a subtype of neutrophils that are found within the TME and exhibit a primary role in promoting tumor growth and metastasis by suppressing effector T lymphocytes and NK cells [15]. In gastric cancer, neutrophils within the TME are more susceptible to ferroptosis compared to those from peripheral blood or normal tissues [16]. However, in the metastatic TME, TINs develop resistance to ferroptosis. Activated by tumor-secreted granulocyte-macrophage colony stimulating factor (GM-CSF) via the downstream JAK/STAT5–CCAAT/enhancer binding protein-β (C/EBPβ) axis, aconitate decarboxylase 1 (Acod1) in TINs produces itaconate, which triggers a nuclear factor erythroid 2-related factor 2 (NRF2)-dependent defense mechanism against ferroptosis. This defense system helps to sustain the persistence of TINs, thus promoting lung metastasis of breast cancer [17]. These findings imply that the microenvironment regulates neutrophil ferroptosis by disrupting iron metabolism and the antioxidant system (as shown in Figure 1a). Generally, neutrophil ferroptosis promotes disease progression, while specific neutrophil subtypes like TINs develop resistance to ferroptosis to exacerbate disease.

### 2.2. Macrophages

Macrophages perform a variety of functions essential for maintaining homeostasis, fighting against infections and regulating immune responses.

Ferroptosis reverses the polarization of tumor-associated macrophages (TAMs) in the TME [18]. TAMs are broadly categorized into pro-inflammatory M1-like and anti-inflammatory M2-like phenotypes, with M1 macrophages exhibiting greater resistance to ferroptosis. This resistance is attributed to NO free radicals (NO•) and inducible nitric oxide synthase (iNOS) produced by M1 cells, which suppress lipid peroxidation by restricting arachidonic acid 15-lipoxygenase-1 (ALOX15) activity [19]. Due to their high plasticity, TAMs can rapidly switch phenotypes in response to TME signals [20]. Ferroptosis promotes the shift from M2 to M1 polarization through the accumulation of ROS and lipid peroxides, which cause DNA damage and activate the cGAS- stimulator of interferon genes (STING)-NF-κB axis, leading to M1 marker expression [21]. In various tumors, apolipoprotein C1 (APOC1) acts as a key suppressor of ferroptosis in TAMs through the GSH/GPX4 axis and the Kelch-like ECH-associated protein 1 (KEAP1)-NRF2 pathway [22,23]. Conversely, dihydroartemisinin (DHA) directly triggers ferroptosis in TAMs [21]. By inhibiting APOC1 or using DHA, TAMs can be effectively reprogrammed into the M1 phenotype through ferroptosis, thereby improving anti-tumor immune responses and immunotherapy efficacy [24].

Iron overload and the subsequent ferroptosis in macrophages accelerate the progression of inflammatory diseases such as atherosclerosis (AS) [25]. Key regulators of iron homeostasis include ferroportin (FPN) for iron export and transferrin receptor (TfR) for iron uptake. *JAK2* promotes macrophage ferroptosis by phosphorylating STAT3 to induce hepcidin transcription [26,27,28]. In addition, cigarette tar upregulates hepcidin through the NF-κB pathway. Elevated hepcidin inhibits FPN, leading to iron overload in macrophages. This accumulation promotes ferroptosis by inhibiting solute carrier family 7 member 11 (SLC7A11/xCT) expression, ultimately accelerating the formation of AS plaques [29,30]. Conversely, the NRF2 pathway suppress macrophage ferroptosis by upregulating SLC7A11 and GPX4, slowing AS progression [31,32,33]. Similarly, inhibiting isocitrate dehydrogenase 1 (IDH1) to reduce oxidized low-density lipoprotein (ox-LDL) level and activate NRF2 can protect macrophages against ferroptosis, preventing the formation of foam cells [34]. Beyond in inflammatory diseases, ferroptosis in macrophages also increases susceptibility to infections. For example, *Mycobacterium tuberculosis* infection induces macrophage and lung tissue necrosis via ferroptosis, and targeted inhibition of this pathway can effectively reduce bacterial load [35]. To summarize, the role of macrophage ferroptosis exhibits duality across different disease contexts. In inflammatory and infectious diseases, it exacerbates tissue damage and disease progression, whereas in tumor, it can enhance anti-tumor immunity by remodeling immune responses. The regulation of macrophage ferroptosis involves a complex interplay of pathways (as shown in Figure 1b), which identifies several key molecules as potential targets for therapeutic intervention.

### 2.3. T Lymphocytes

T lymphocytes are a vital component of the adaptive immune response. They are categorized into several types, mainly including cytotoxic CD8^+^ T cells, helper CD4^+^ T cells and regulatory T cells (Tregs).

#### 2.3.1. CD8^+^ T Cells

CD8^+^ T cells or cytotoxic T cells are capable of specifically recognizing and directly eliminating infected and cancer cells primarily through the production and release of perforin and granzymes [36].

However, their efficacy is critically undermined within the TME, where CD8^+^ T cells are more susceptible to ferroptosis due to nutrient deficiencies and metabolic stress [37]. A main alteration in the TME is the accumulation of lipid. Cholesterol enrichment in the TME upregulates CD36 on CD8^+^ T cells. CD36-mediated fatty acid uptake subsequently triggers lipid peroxidation and ferroptosis, ultimately impairing anti-tumor activity [38]. Furthermore, CD36-mediated uptake of ox-LDL by CD8^+^ T cells can activate the p38 kinase pathway, which is important in stress responses and inflammation [39].

**Figure 2:**
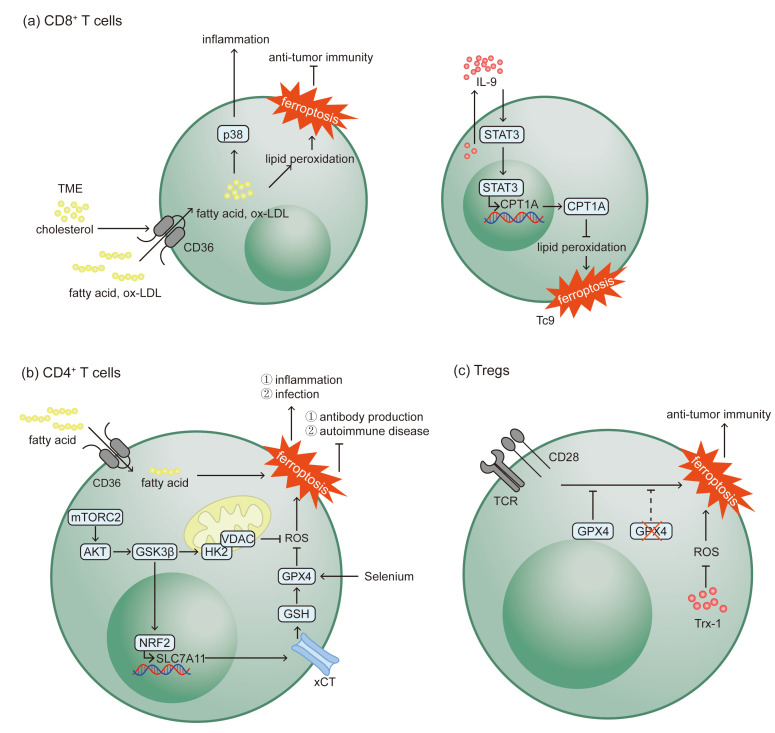
Ferroptosis mechanisms and consequences of T lymphocytes. (**a**) Increased uptake of lipid by CD36 triggers ferroptosis in CD8^+^ T cells and activates the p38 kinase pathway, impairing anti-tumor immunity and promoting inflammatory response. Tc9 cells display resistance to ferroptosis via the IL-9/STAT3/CPT1A axis. (**b**) Elevated CD36-mediated uptake of fatty acid trigger ferroptosis in CD4^+^ T cells. Upregulated GPX4 and mTORC2 enable CD4^+^ T cells to resist ferroptosis. (**c**) GPX4 and Trx-1 help Tregs to avert ferroptosis, while GPX4-deficient Tregs undergo lipid peroxidation and ferroptosis, thereby bolstering anti-tumor immunity (the “×” symbol on GPX4 indicates GPX4-deficient conditions).

Notably, this susceptibility is not uniform across all CD8^+^ T cells. Among the various subsets, the interleukin (IL)-9-secreting cytotoxic T lymphocyte subset (Tc) 9 exhibits remarkable resistance to ferroptosis. Through the IL-9/STAT3 axis, Tc9 subset upregulates carnitine palmitoyltransferase I (CPT1A), enhancing fatty acid oxidation to deplete lipid substrates within the cells. As a result, these cells effectively resist ferroptosis and potent stronger anti-tumor immunity compared to the Tc1 subset [40]. These findings indicate that ferroptosis susceptibility is not fixed but determined by the differentiation state and metabolic programming of CD8^+^ T cells (as shown in Figure 2a). Given the differences in ferroptosis susceptibility among CD8^+^ T cell subsets, future therapeutic strategies, such as cell therapies targeting tumors, should not only focus on the cytotoxic function of T cells but also take into account their metabolic characteristics and survival capabilities.

#### 2.3.2. CD4^+^ T Cells

CD4^+^ T cells or T helper (Th) cells are crucial in adaptive immunity. Naive CD4^+^ T cells differentiate into various effector lineages, such as Th1, Th2, Th17 and follicular helper T (Tfh) cells [41].

Similarly to CD8^+^ T cells, CD36-mediated lipid uptake also promotes ferroptosis in CD4^+^ T cells, contributing to immune dysfunction [42]. After trauma, downregulation of the xCT-GSH-GPX4 axis induces ferroptosis and subsequent exhaustion of CD4^+^ T cells, raising the risk of sepsis [43]. Tfh cells, essential for antibody production and influenza vaccine responses, are highly vulnerable to ferroptosis due to T cell receptor (TCR)-induced mitochondrial ROS. GPX4, as the primary antioxidant against ferroptosis, is essential for the survival of CD4^+^ T cells [44]. Beyond GPX4, CD4^+^ T cells have alternative pathways to avoid ferroptosis. For example, the mammalian target of rapamycin complex 2 (mTORC2)–AKT–glycogen synthase kinase 3β (GSK3β) axis reduces mitochondrial ROS by strengthening Hexokinase 2 (HK2)–voltage-dependent anion channel (VDAC) interaction. It also activates NRF2 to sustain GPX4 activity, thereby protecting virus-specific memory CD4^+^ T cells from ferroptosis and microbial reinfection [45]. Ferroptosis in CD4^+^ T cells compromises immune response and causes inflammatory imbalance. Supplementing selenium can increase GPX4 levels and help prevent ferroptosis [44].

In contrast, in multiple sclerosis (MS) and its experimental autoimmune encephalomyelitis (EAE) model, CD4^+^ T cells upregulate GPX4 to evade ferroptosis. This enhances their survival and inflammatory function, thereby accelerating disease progression [46]. Collectively, these findings demonstrate the complex regulation and outcomes of ferroptosis in CD4^+^ T cells in inflammatory progress (as shown in Figure 2b).

#### 2.3.3. Regulatory T Cells

As a specific subgroup of T lymphocytes, Tregs exhibit strong immunosuppressive ability. They act as key mediators of immune tolerance and tumor evasion mainly by repressing distinct T cell responses [47].

ROS-induced oxidative stress exacerbates the production of lipid peroxidation. A main feature of the TME is the continued oxidative stress. Previous studies found that Tregs are more tolerant to oxidative stress-induced cell death than effector T lymphocytes due to its ability to express and secrete higher levels of thioredoxin-1 (Trx-1), a major antioxidative molecule [48]. Xu et al. found that GPX4 helped Tregs to avert ferroptosis in response to the co-stimulation signal of TCR/CD28. In contrast, GPX4-deficient Tregs underwent lipid peroxidation and ferroptosis, reducing their suppressive function and increasing the activity of other immune cells like CD8^+^ T cells and DCs due to the release of IL-1β. Consequently, this enhancement in immune cell activity contributes to the limitation of tumor growth and the bolstering of anti-tumor immunity [49]. Hence, antioxidative molecules are crucial for ferroptosis resistance in Tregs (as shown in Figure 2c) and represent a therapeutic target for anticancer strategies.

### 2.4. B Lymphocytes

B lymphocytes are categorized into various subclasses based on developmental origin and functional characteristics, including innate-like cells such as B1 and marginal zone B lymphocytes, and follicular B2 cells. Each subclass has unique requirements for GPX4 to eliminate lipid ROS and prevent ferroptosis, due to their distinct lipid metabolism. GPX4 is indispensable for the development, homeostatic maintenance and antibody response of B1 and marginal zone B lymphocytes due to their high expression of fatty acid transport proteins like CD36 [50].

**Figure 3:**
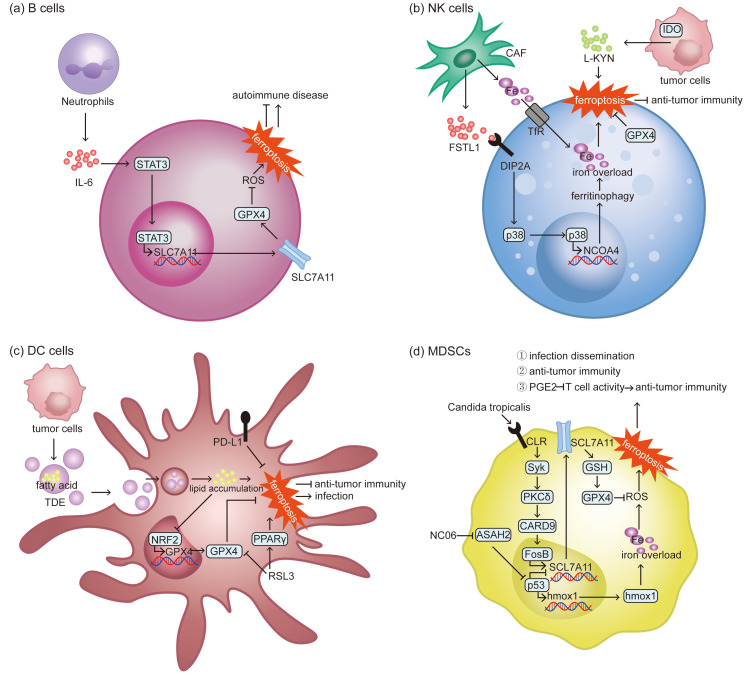
Ferroptosis mechanisms and consequences of B lymphocytes, NK cells, DCs and MDSCs. (**a**) GPX4 is essential for B1 and marginal zone B lymphocytes to resist ferroptosis. Ferroptosis of B cells plays a dual role in autoimmune diseases. (**b**) Various components in the TME induce ferroptosis of NK cells, thereby impairing anti-tumor immunity. (**c**) Within the TME, TDE lipids and PPARγ activation promote DC ferroptosis, whereas PD-L1 exerts a protective effect to sustain immune responses. In sepsis, Sestrin2 alleviates LPS-triggered DC ferroptosis, maintaining immune homeostasis. (**d**) *Candida* infection promotes ferroptosis in MDSCs, which results in disseminated infection. In tumors, MDSCs upregulate ASAH2 to block ferroptosis and maintain immunosuppression. MDSC ferroptosis shows dual effects on anti-tumor immunity.

Systemic lupus erythematosus (SLE) is a chronic autoimmune disease characterized by dysregulated activation of B cells and the production of autoantibodies. Ferroptosis in B cells is closely related to the pathogenesis of SLE. Chen et al. demonstrated that peripheral B cells in SLE are prone to undergo ferroptosis, which promotes their differentiation into plasma cells, enhances autoantibody production and exacerbates damage to organs like kidney. Inhibition of ferroptosis significantly alleviated disease activity and renal pathology, highlighting ferroptosis as a driver of SLE progression [51]. However, within the lupus kidney microenvironment, Wang et al. identified a subset of neutrophils that highly express IL-6. These neutrophils activate the IL-6/STAT3 pathway to upregulate SLC7A11 in B cells. This enhances their antioxidant capacity and resistance to ferroptosis. In this context, inhibiting SLC7A11 significantly enhanced B cell ferroptosis and reduced pathological B cell proliferation, decelerating the development of lupus nephritis [52]. The opposite conclusions from these two studies likely arise from differences in the sources of B cells examined and their microenvironments. Distinct microenvironments significantly influence the regulatory mechanisms of ferroptosis in B cells and their ultimate role in disease progression (as shown in Figure 3a). Therefore, future studies should prioritize a more detailed analysis of ferroptosis across different B cell subtypes and microenvironments, which will facilitate the design of targeted treatments for autoimmune conditions like SLE.

### 2.5. Natural Killer Cells

NK cells are crucial for innate immunity. It lyses pathogens by generating cytotoxic molecules like perforin and granzyme B and activates the immune system by secreting immune factors like IFN-γ [53]. Their immune response is regulated by activating or inhibitory signals originating from the membrane. This comes from both direct cell-to-cell contact and the release of soluble factors. Despite great advancements has been made in the study of NK cells, the efficacy of NK-based immunotherapy is still limited in cancer especially in solid tumors [54].

NK cells in TME display features of ferroptosis accompanied by lipid peroxidation and oxidative damage, which suppresses glucose metabolism in NK and hinders their cytotoxicity, damaging anti-tumor immunity [55]. Stromal components and tumor cells within the TME may induce ferroptosis in NK cells (as shown in Figure 3b) [56]. Cancer-associated fibroblasts (CAFs), a primary stroma component in the TME, contribute to an immunosuppressive TME that favors tumor progression and immune evasion [57]. They are capable of disrupting iron homeostasis, leading to ferroptosis in NK cells and subsequent defects in their function. Yao et al. found in gastric cancer that CAFs exported iron to the TME, leading to iron overload in NK cells. Meanwhile, CAFs-derived follistatin like protein 1 (FSTL1) improves the expression of nuclear receptor coactivator 4 (NCOA4), a key molecule involved in ferritinophagy, in the NK cells through disco interacting protein 2 homolog A (DIP2A)-p38 pathway [58]. In addition, GC cells produce L-kynurenine (L-KYN) in the presence of enzyme indoleamine 2,3-dioxygenase (IDO), which induces ferroptosis in NK cells in an aryl hydrocarbon receptor (AHR)-independent manner, leading to a reduction in NK cell infiltration. However, NK cells with high level of GPX4 can resist L-KYN induced ferroptosis [59]. These discoveries inspire us that exploring the relation between NK ferroptosis and TME may provide strategies to enhance the efficacy of NK-based immunotherapy.

### 2.6. Dendritic Cells

DCs are the most potent antigen-presenting cells that bridge innate and adaptive immune response by activating T lymphocytes.

Recent studies have found that ferroptosis in DCs has become a key mechanism of tumor immune escape, with lipid metabolism disorders as a central contributing factor. Tumor-derived exosomes (TDEs) are a kind of vesicle transferring substances like nucleic acids, proteins and fatty acids to nearby or distant cells, which regulates immune response [60]. Ferroptotic DCs induced by TDEs show severe functional defects. These cells fail to mature properly, produce key cytokines, or present antigens via major histocompatibility complex (MHC) molecules effectively. As a result, they cannot activate T cells, leading to widespread immunosuppression. Yang et al. reported that exosomes derived from Glioblastoma led to lipid accumulation in DCs through the direct transfer of fatty acid, and NRF2/GPX4 pathway gets involved in this ferroptosis process [61].

In addition, peroxisome proliferator-activated receptor gamma (PPARγ) is a ligand activated transcription factor that determines lipid metabolism in human diseases. Han et al. found that RSL3, a GPX4 inhibitor, induces PPARγ-dependent ferroptosis in DCs, which severely compromise anti-tumor immunity. However, it is found that SLC7A11 has a low expression in DCs and it is the GPX4 inhibitor RSL3 not the SLC7A11 inhibitor erastin triggers ferroptosis in DCs, indicating that the activation of GPX4 in DCs may not depend on SLC7A11 [62]. Therefore, further studies should be made to explore other pathways regulating GPX4 in DCs.

However, protective mechanisms against ferroptosis also exist. High expression of PD-L1 helps DC to resist chemotherapy-induced ferroptosis. PD-L1 deficiency downregulates SLC7A11, increasing ferroptosis susceptibility and thereby weakening chemotherapy efficacy [63]. In addition, in infectious diseases such as sepsis, lipopolysaccharide (LPS) induces ferroptosis in DCs, while Sestrin2 inhibits this process through the ATF4-C/EBP homologous protein (CHOP)-cation transport regulator homolog 1 (CHAC1) signaling pathway, thereby alleviating sepsis [64]. Overall, in tumors and infectious diseases, DCs often undergo ferroptosis due to abnormal lipid metabolism, promoting disease progression, and the use of small molecule inhibitors can suppress DC ferroptosis (as shown in Figure 3c).

### 2.7. MDSCs

In response to inflammation, the generation and activation of myeloid cells are indeed a critical part of the immune response. However, prolonged inflammation disrupts the normal differentiation and maturation of myeloid cells, leading to the accumulation of immature immunosuppressive myeloid cells, primarily MDSCs [65]. MDSCs exhibit potent suppressive activities against effector lymphocytes and play a crucial role in the regulation of immune responses, particularly in the context of cancer and infectious diseases [66,67].

In cases of disseminated *Candida tropicalis* infection, the recognition of *Candida tropicalis* by C-type lectin receptors stimulates the spleen tyrosine kinase (Syk)–protein kinase C-δ (PKCδ)–Caspase Recruitment Domain-containing Protein 9 (CARD9)–FosB signaling pathway. CARD9 is crucial for antifungal immunity. CARD9 deficiency reduces SLC7A11 expression in the kidneys and promote the ferroptosis of MDSCs, which results in disseminated *Candida tropicalis* infection and aggravated kidney damage [68]. The activation of p53 represents another pathway to regulate ferroptosis, also by reducing SLC7A11 gene transcription [69]. In colon carcinoma, tumor-infiltrating MDSC exhibits resistance to ferroptosis by the high expression of N-acylsphingosine amidohydrolase (ASAH2), a kind of neutral ceramidase, which suppresses the p53-heme oxygenase 1 (hmox-1) signaling pathway. Targeting ASAH2 with NC06 to induce ferroptosis in MDSCs could be a promising therapeutic strategy to suppress MDSCs accumulation and enhance the efficacy of cancer immunotherapy [70].

Polymorphonuclear myeloid-derived suppressor cells (PMN-MDSCs) are a subset of MDSCs. Hypoxia-mediated downregulation of GPX4 in tumor PMN-MDSC renders it susceptible to ferroptosis. Contrary to the general belief that decreasing PMN-MDSCs abundance diminished their immunosuppressive capacity, Kim et al. discovered that ferroptosis triggered the release of oxygenated lipids like prostaglandin E2 (PGE2) and restricted T cell proliferation and activity, thereby establishing an immunosuppressive microenvironment that favors tumor growth [71]. These findings reveal the diversity of MDSC ferroptosis mechanisms and their immune consequences (as shown in Figure 3d). When assessing the impact of immune cell ferroptosis on disease progression, it is essential to consider not only the ferroptotic cells but also their regulatory effects on other immune cells within the microenvironment.

## 3. Ferroptosis-Based Interventions

**Figure 4:**
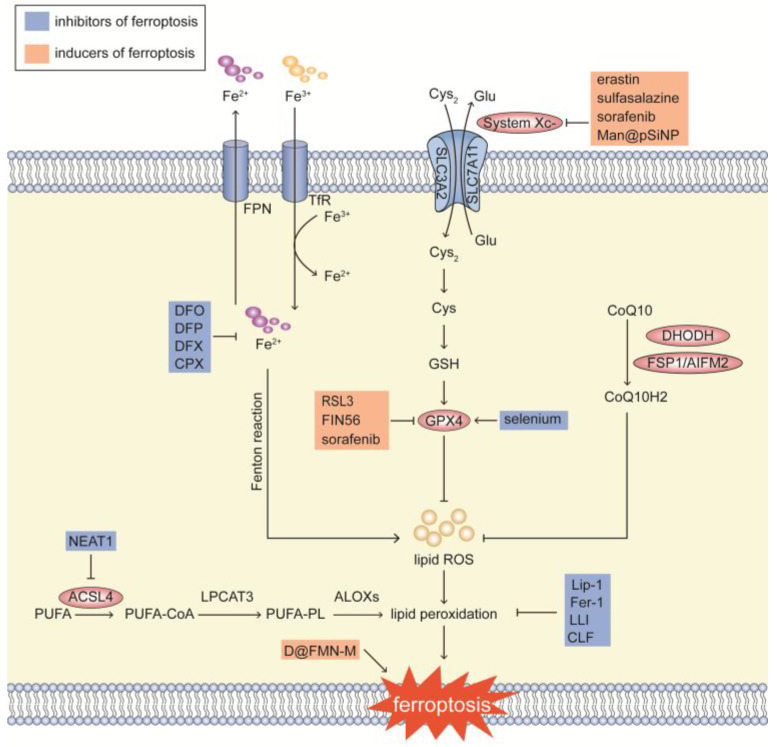
Ferroptosis-based interventions. Strategies to induce ferroptosis mainly involve inhibiting GPX4 or disrupting system Xc^−^. In contrast, ferroptosis can be suppressed by reducing intracellular iron levels, weakening antioxidant defenses, or blocking lipid peroxidation. Nanomedicine-based approaches can specifically regulate ferroptosis in immune cells.

Due to its substantial therapeutic potential, ferroptosis has garnered great attention in disease treatment. With the discovery of its mechanisms and key targets, considerable progress has been made in developing pharmacological agonists and antagonists for the treatment of these ferroptosis-related conditions (as shown in Figure 4).

### 3.1. Small-Molecule Ferroptosis Inducers

Recent studies have identified four main mechanisms that induce ferroptosis and various inducers have been developed to target these pathways. First, blocking GPX4 is a key approach. Compounds like RSL3, FIN 56 and sorafenib can inhibit GPX4, thereby promoting ferroptosis [72,73]. Second, inhibiting the glutamate-cystine transport system (System Xc^−^) is another effective strategy. Compounds such as erastin and its derivatives, sulfasalazine and sorafenib are known to disrupt this system, leading to ferroptosis [74]. Third, inhibiting the reduction of CoQ10 through the targeting of apoptosis-inducing factor mitochondria-associated 2 (AIFM2)/FSP1 or dihydroorotate dehydrogenase (DHODH) can also induce ferroptosis. Finally, inducing lipid peroxidation through the use of peroxides, iron, or PUFA overload is another mechanism that leads to ferroptosis [75]. However, the application of non-specific ferroptosis inducers requires careful consideration due to their potential off-target effects. For instance, in cancer, the efficacy of such inducers has been largely confined to immunodeficient animal models, as they may not only eliminate tumor cells but also trigger ferroptosis in immune cells, undermining the anti-tumor response [71].

As discussed in previous sections, certain immune cells acquire ferroptosis resistance during disease progression. Targeting these cells with ferroptosis inducers thus represents a promising treatment. NC06 is a selective ASAH2 small molecule inhibitor that specifically targets immunosuppressive MDSCs in the tumor microenvironment, inducing ferroptosis in them without directly causing ferroptosis in tumor cells. This mechanism alleviates immunosuppression, enhances anti-tumor T-cell responses and ultimately inhibits tumor growth [70]. In addition to studies on tumor, in SLE, Wu et al. discovered that CX-5461, a selective RNA polymerase I inhibitor, specifically triggers ferroptosis in B cells through the p53-SLC7A11-ALOX12 pathway and treats SLE without any pathogenic effects on vital organs [76].

However, ferroptosis inducers carry potential risks as well. For example, inducing ferroptosis in Tregs enhances anti-tumor immunity, but it may provoke the release of proinflammatory factors like IL-1β, which could promote T_H_17 responses and increase potential autoimmune risks. This emphasizes the need for combination strategies, such as coupling ferroptosis inducers with IL-1β blockade, to minimize potential adverse effects and improve therapeutic safety [49].

### 3.2. Small-Molecule Ferroptosis Inhibitors

Current efforts to inhibit ferroptosis can be divided into (a) controlling iron availability, like iron chelator deferoxamine (DFO), deferiprone (DFP), deferasirox (DFX) and ciclopirox (CPX) which can reduce iron levels [77,78,79,80], (b) directly blocking this peroxidation process, like ferrostatin-1 (Fer-1) and liproxstatin-1 (Lip-1) [81], (c) inhibiting generation of the substrate lipids for peroxidation through the action of lipid biosynthesis and ACSL4, like nuclear paraspeckle assembly transcript 1 (NEAT1) [82], and (d) boost the GSH/GPX4 axis, like selenium [44]. However, the development of targeted small-molecule ferroptosis inhibitors remains a significant challenge and most of these interventions are administered systemically rather than in a cell-specific manner. Existing strategies often involve either pretreating specific immune cells in vitro with iron chelators, Fer-1 or Lip-1, or administering these compounds in vivo via intraperitoneal injection. For example, in a model of tuberculosis, systemic Fer-1 treatment not only reduces lung necrosis but also decreases bacterial loads in the lungs and spleens of infected mice [35]. Similarly, in EL-4 and LLC TB models, intraperitoneal injection of Lip-1 abolishes the immunosuppressive function of PMN-MDSCs, resulting in reduction in tumor growth [71].

### 3.3. Nanomaterials

Traditional ferroptosis agents face limitations such as poor bioavailability and low stability. Nanomaterials carrying bioactive components offer a promising alternative by improving delivery efficiency and specificity. These systems enhance uptake, reduce toxicity and enable targeted therapy [83,84]. Current research mainly focuses on macrophage-targeted applications, leaving other immune cells underexplored. This section will review advances in nanomaterial-mediated ferroptosis regulation in macrophages.

Developing biomimetic ferroptosis inducers is an innovative approach, and D@FMN-M is one such inducer. It is fabricated by loading DHA in Fe-HMSNs and modifying it with bacterial outer membrane vesicles. D@FMN-M induced dual ferroptosis in both tumor cells and TAMs by generating abundant free radicals. It also converted M2 macrophages into M1 macrophages, thereby reversing immune tolerance and enhancing immunotherapeutic efficacy [85]. Furthermore, erastin-loaded mannose-functionalized porous silicon nanoparticles (Man@pSiNPs) have been designed to specifically target TAMs, achieving intracellular ferroptosis activation and regulated TAM phenotype. The combination of Man@pSiNPs with anti-PD-L1 treatment has demonstrated optimal anticancer efficacy. This synergistic effect may be attributed to the ability of ferroptosis to enhance the immunogenicity of dying tumor cells, thereby amplifying the response to immune checkpoint blockade therapies [86]. However, overactivation of ferroptosis in macrophages can trigger massive cell death, which compromises their antigen-presentation function and may worsen immunosuppression. It could also induce excessive inflammatory reactions, leading to potential tissue damage or autoimmune disorders.

The advent of nanomaterials also makes it possible to combine ferroptosis with other therapeutic approaches such as photodynamic therapy (PDT). PDT employs photosensitizers activated by light to amplifies ROS generation and induces immunogenic cell death (ICD). A recent study developed an innovative nanodrug LLI by encapsulating ferroptosis inhibitor Lip-1 and photosensitizer di-iodinated IR780 (Icy7) within liposomes to inhibit neutrophil ferroptosis and induced the ICD of tumor cells in gastric cancer. This synergistic approach also boosted the effectiveness of anti-PD-1 treatment [16].

Nanomaterials designed to target macrophages are also utilized in the treatment of inflammatory diseases. In acidic inflammatory environment, a calcium-carbonate (CaCO_3_) mineralized liposome (CaCO_3_@Lipo@Fer-1, CLF) encapsulating Fer-1 released Ca^2+^, which promoted M2 polarization via the calcium-sensing receptor (CaSR)/AKT/β-catenin pathway. Meanwhile, the released Fer-1 effectively inhibited ferroptosis in M2 macrophages, thus improving the therapeutic effect of inflammatory bowel disease in vivo [87]. In conclusion, using nanomaterials to enhance targeted ferroptosis induction and inhibition offers a promising avenue for improved disease treatment.

## 4. Perspectives

Ferroptosis has attracted widespread attention since its discovery in 2012. Great advancement has been made in understanding its physiological functions, mechanisms and potential therapeutic applications. Ferroptosis is driven by iron-dependent lipid peroxidation and regulated by multiple metabolic and signal pathways related to diseases.

In infection, inflammation and cancer, immune effector cells such as neutrophils, macrophages, T cells, B cells, DCs and NK cells are often highly susceptible to ferroptosis. This susceptibility weakens the immune response and exacerbates disease progression. In contrast, immunosuppressive cells like TINs, Tregs and MDSCs frequently avoid ferroptosis by enhancing their antioxidant defenses mainly via GPX4. This allows them to continue suppressing immune activity. Current evidence highlights that ferroptosis affects immune cells differently based on the disease context. Interestingly, this pattern may be reversed in autoimmune disorders, where overactivated immune cells like CD4^+^ T cells and B cells that resist ferroptosis may exhibit enhanced pathogenicity and contribute to tissue damage.

Although targeting ferroptosis has emerged as a promising therapeutic strategy, most current approaches remain narrowly focused. Many studies investigate ferroptosis modulation of a specific immune cell type without systematically evaluating the broader impact on the immune network. Additionally, current research on ferroptosis-targeted therapies rely heavily on in vitro cell lines and in vivo mouse models, which poorly replicate the human microenvironment.

The non-specific application of ferroptosis inducers or inhibitors may lead to inconsistent treatment outcomes and cause off-target toxicity or immune-related adverse effects by disrupting the balance between immune cells. However, therapies specifically targeting immune cells may also have limitations due to excessive impairment of normal immune functions or triggering excessive inflammatory responses.

Therefore, future research should prioritize understanding the influences of ferroptosis on different immune cells and the whole immune environment. More human-relevant models, such as organoids and humanized systems, should be employed to validate these findings and advance these strategies toward clinical applications. Meanwhile, developing methods to target specific immune cells or combination treatments may help achieve safer and more effective therapies for infections, inflammatory diseases and tumors.

## Data Availability

Not applicable.

## Abbreviations

The following abbreviations are used in this manuscript:

Acod1	aconitate decarboxylase 1
ACSL4	acyl-coenzyme A synthetase long-chain family member 4
AHR	aryl hydrocarbon receptor
AIFM2	apoptosis-inducing factor mitochondria-associated 2
ALOX15	arachidonic acid 15-lipoxygenase-1
APOC1	apolipoprotein C1
AS	atherosclerosis
ASAH2	N-acylsphingosine amidohydrolase
BH4	tetrahydrobiopterin
C/EBPβ	CCAAT/enhancer binding protein-β
CaCO3	calcium-carbonate
CAFs	cancer-associated fibroblasts
CaMKIV	calcium/calmodulin kinase IV
CARD9	Caspase Recruitment Domain-containing Protein 9
CaSR	calcium-sensing receptor
CHAC1	cation transport regulator homolog 1
CHOP	C/EBP homologous protein
CoQ10	Coenzyme Q10
CPT1A	carnitine palmitoyltransferase I
CPX	ciclopirox
CREMα	cAMP-responsive element modulator α
DCs	dendritic cells
DFO	deferoxamine
DFP	deferiprone
DFX	deferasirox
DHA	dihydroartemisinin
DHODH	dihydroorotate dehydrogenase
DIP2A	disco interacting protein 2 homolog A
EAE	experimental autoimmune encephalomyelitis
Fer-1	ferrostatin-1
FPN	ferroportin
FSP1	ferroptosis suppressor protein 1
FSTL1	follistatin like protein 1
GCH1	GTP cyclohydrolase-1
GM-CSF	granulocyte-macrophage colony stimulating factor
GPX4	glutathione peroxidase 4
GSH	glutathione
GSK3β	glycogen synthase kinase 3β
HK2	hexokinase 2
hmox-1	heme oxygenase 1
ICD	immunogenic cell death
IDH1	isocitrate dehydrogenase 1
IDO	indoleamine 2,3-dioxygenase
IFN	interferon
IL	interleukin
iNOS	inducible nitric oxide synthase
KEAP1	Kelch-like ECH-associated protein 1
Lip-1	liproxstatin-1
L-KYN	L-kynurenine
LPCAT3	lysophosphatidylcholine acyltransferase 3
LPS	lipopolysaccharide
MDSCs	myeloid-derived suppressor cells
MHC	major histocompatibility complex
MS	multiple sclerosis
mTORC2	mammalian target of rapamycin complex 2
NCOA4	nuclear receptor coactivator 4
NEAT1	nuclear paraspeckle assembly transcript 1
NETs	neutrophil extracellular traps
NK	natural killer
NO•	nitric oxide free radical
NRF2	nuclear factor erythroid 2-related factor 2
ox-LDL	oxidized low-density lipoprotein
PDT	photodynamic therapy
PGE2	prostaglandin E2
PKCδ	protein kinase C-δ
PMN-MDSCs	polymorphonuclear myeloid-derived suppressor cells
PPARγ	peroxisome proliferator-activated receptor gamma
PUFAs	polyunsaturated fatty acids
ROS	reactive oxygen species
SLC7A11	solute carrier family 7 member 11
SLE	systemic lupus erythematosus
STING	stimulator of interferon genes
Syk	spleen tyrosine kinase
TAMs	tumor-associated macrophages
Tc	cytotoxic T lymphocyte subset
TCR	T-cell receptor
TDEs	tumor-derived exosomes
Tfh	follicular helper T
TfR	transferrin receptor
Th	T helper
TINs	Tumor-infiltrating neutrophils
TME	tumor microenvironment
Tregs	regulatory T cells
Trx-1	thioredoxin-1
VDAC	voltage-dependent anion channel
